# Localization of QTLs for *in vitro *plant regeneration in tomato

**DOI:** 10.1186/1471-2229-11-140

**Published:** 2011-10-20

**Authors:** Carlos Trujillo-Moya, Carmina Gisbert, Santiago Vilanova, Fernando Nuez

**Affiliations:** 1 Instituto de Conservación y Mejora de la Agrodiversidad Valenciana (COMAV) Universitat Politècnica de València, Camino de Vera, 14 46022 Valencia, Spain

## Abstract

**Background:**

Low regeneration ability limits biotechnological breeding approaches. The influence of genotype in the regeneration response is high in both tomato and other important crops. Despite the various studies that have been carried out on regeneration genetics, little is known about the key genes involved in this process. The aim of this study was to localize the genetic factors affecting regeneration in tomato.

**Results:**

We developed two mapping populations (F_2 _and BC_1_) derived from a previously selected tomato cultivar (cv. Anl27) with low regeneration ability and a high regeneration accession of the wild species *Solanum pennellii *(PE-47). The phenotypic assay indicated dominance for bud induction and additive effects for both the percentage of explants with shoots and the number of regenerated shoots per explant. Two linkage maps were developed and six QTLs were identified on five chromosomes (1, 3, 4, 7 and 8) in the BC_1 _population by means of the Interval Mapping and restricted Multiple QTL Mapping methods. These QTLs came from *S. pennellii*, with the exception of the minor QTL located on chromosome 8, which was provided by cv. Anl27. The main QTLs correspond to those detected on chromosomes 1 and 7. In the F_2 _population, a QTL on chromosome 7 was identified on a similar region as that detected in the BC_1 _population. Marker segregation distortion was observed in this population in those areas where the QTLs of BC_1 _were detected. Furthermore, we located two tomato candidate genes using a marker linked to the high regeneration gene: *Rg-2 *(a putative allele of *Rg-1*) and *LESK1*, which encodes a serine/threonine kinase and was proposed as a marker for regeneration competence. As a result, we located a putative allele of *Rg-2 *in the QTL detected on chromosome 3 that we named *Rg-3*. *LESK1*, which is also situated on chromosome 3, is outside *Rg-3*. In a preliminary exploration of the detected QTL peaks, we found several genes that may be related to regeneration.

**Conclusions:**

In this study we have identified new QTLs related to the complex process of regeneration from tissue culture. We have also located two candidate genes, discovering a putative allele of the high regeneration gene *Rg-1 *in the QTL on chromosome 3. The identified QTLs could represent a significant step toward the understanding of this process and the identification of other related candidate genes. It will also most likely facilitate the development of molecular markers for use in gene isolation.

## Background

*In vitro *regeneration of cultivated tomato (*Solanum lycopersicum *L.) has been a constant subject of research because of the commercial value of the crop. Consequently, numerous studies on plant regeneration from a wide range of tissues and organs of wild and cultivated tomato germplasm have been published [1]. These studies demonstrate that organogenesis, the common tomato regeneration pathway, is strongly influenced by genotype as well as by several physical and chemical factors. These reports also document the existence of recalcitrance (partial or total inability to respond to *in vitro *culture), which greatly limits biotechnological breeding. High regeneration is crucial to the success of techniques such as haploid regeneration, genetic transformation, propagation, somatic hybridization, mutation selection and germplasm storage [2,3]. For example, the low efficiency of tomato transformation has been associated with the low regeneration potential of the cultivars used [4,5]. In addition, in some cultivars, buds may be induced but do not develop into shoots [6]. In order to increase regeneration ability in low regenerating tomato cultivars, several introgression programs have been documented [7-10].

The process of *in vitro *shoot organogenesis usually involves a hormonal response of somatic cells, the dedifferentiation of differentiated cells in order to acquire organogenic competence, cell division of the responding cell(s) and initiation and development of new shoots from the newly dividing cell(s), either directly or indirectly through a callus stage [11,12]. Thus, many genes may be involved at different steps of this complex process. For instance, the *cdc2 *gene expression, which encodes p34, a key cell cycle regulator, has been proposed as an indicator of the state of competence to divide [13]. Genes that encode or regulate cytokinins and auxin may clearly influence regeneration. Both types of growth regulators act synergistically to promote cell division and antagonistically to promote shoot and root initiation from callus cultures [14]. In *Arabidopsis*, a Histidine Kinase (AHK) gene that encodes a cytokinin receptor (CRE1/AHK4) has been identified [15,16] and linked, like other AHKs, to cell division and regulation [17]. With regard to the initiation of shoot formation, the most characterized gene reported is *ESR1*, which confers, when overexpressed, cytokinin-independent shoot formation in *Arabidopsis *root explants [18]. *ESR1 *encodes a transcription factor belonging to the ethylene-responsive factor (ERF) family and is classified in subgroup VIII-b. The *ESR2 *gene that encodes a protein that is very similar to *ESR1 *appears to have redundant functions that regulate shoot regeneration [19]. The expression patterns of other Arabidopsis ERF VIII-b subgroup genes may also be involved in early events of shoot regeneration [20].

Genetic analysis of regeneration in tomato suggests that dominant alleles determine high regeneration capacity [7,21-24]. However, there is no consensus about the number of genes involved. For instance, Koorneef et al. [25] obtained regeneration segregation ratios in accordance with either a monogenic, digenic or trigenic model depending on the tester tomato line, despite the fact that none of the lines themselves were able to regenerate shoots from root explants. In this study, a dominant allele of *S. peruvianum *L. (*Rg-1*), which determines efficient shoot regeneration in tomato root explants, was mapped near the middle of chromosome 3. In addition, a putative allele of *Rg-1 *from *S*. *chilense *(Dunal) Reiche (*Rg-2*) was reported by Takashina et al. [9] and Satoh et al. [22]. Both alleles may act in combination with other alleles of either tomato or the wild relatives *S. peruvianum *or *S. chilense *[22,25]. On the other hand, Torelli et al. [26] identified a cDNA by mRNA-differential display that corresponded to the *LESK1 *gene and whose expression is specifically and transiently enhanced by the exposure to the hormonal treatment leading to caulogenesis (shoot induction). This gene encodes a putative serine-threonine kinase and has been reported as an *in vitro *caulogenesis marker in tomato [27,28].

Despite ongoing research into the genetic control of *in vitro *culture traits in tomato and other crops, there is still not enough information regarding which key genes are responsible for low or high regeneration ability, nor even the number of genes involved. The study and characterization of the reported genes and others that might be identified could greatly improve our understanding of the molecular mechanism underlying the different phases of tomato *in vitro *organogenesis. In the present study, we developed two mapping populations (F_2 _and BC_1_) from *S. lycopersicum *(as the recurrent parent) and *S. pennellii *Correll (as the regenerating parent) and conducted a QTL-based analysis. We hereby report the identification of six QTLs on five chromosomes. These QTLs present high significant LOD scores and together represent a high percentage of phenotypic variance. We also report markers associated with QTL peaks. In addition, we located two candidate genes, *Rg-2 *and *LESK1*, and performed a preliminary search for genes situated at QTL peaks. Our findings will complement the current knowledge of the genetics of regeneration and facilitate the development of molecular markers for use in tomato breeding and gene isolation.

## Results

### Development of populations and evaluation of the regeneration ability

Two mapping populations, F_2 _and BC_1_, were obtained from a low regenerating cultivar of tomato (cv. Anl27) and the organogenic accession of *S. pennellii *(PE-47). The BC_1 _population was obtained using the tomato cultivar as the recurrent parent. In the first assay, the regeneration ability of the parents and the F_1 _plant used for obtaining the mapping populations was checked by culturing leaf explants on shoot induction medium. Regeneration occurred with little callus development and can be considered as direct. As expected, *S. pennellii *and F_1 _explants manifested a higher regeneration potential versus *S. lycopersicum *explants (*P <*0.001). The percentage of explants with buds (B) in *S. pennellii *was 100%, whereas only 10% was obtained in tomato cv. Anl27 (Table 1). Data obtained in F_1 _for B do not significantly differ from those obtained for *S. pennellii*. The percentage of explants with shoots (R) and the number of regenerated plants per explant with shoots, considered to be the productivity rate (PR), was also higher in *S. pennellii *and F_1 _than in cv. Anl27. However, for these traits (R and PR), the F_1 _values differ significantly from those of *S. pennellii *(Table 1).

**Table 1:** Phenotyping parental genotypes and mapping population

	First Assay Phenotyping parental genotypes and F_1_			Second Assay Phenotyping mapping populations		
	**B^a, c^**	**R^a, c^**	**PR^b, c^**	**B^a, c^**	**R^a, c^**	**PR^b, c^**

*S. pennellii*	100 b	96 c	6.36 c	100.00 c	95.00 d	6.74 c
*S. lycopersicum*	10 a	6 a	0.30 a	7.50 a	2.50 a	0.12 a
F1	90 b	78 b	3.17 b	87.50 c	70.0 c	3.08 b
F_2_	-	-	-	76.91 bc	63.92 c	2.65 b
BC_1_	-	-	-	59.48 b	36.65 b	1.67 b

Means of the traits: percentage of explants with buds (B), percentage of explants with shoots (R) and number of shoots per explant with shoots (PR) for the parent genotypes (*S. pennellii *and *S. lycopersicum*), F_1_, F_2 _and BC_1_.

^a ^B and R are the percentages of explants able to develop buds and shoots, respectively. ^b ^PR is the number of shoots per explant with shoots. ^c ^Mean values within a column separated by different letters are significantly different (*P *< 0.05) according to Duncan's multiple range test.

**Figure 1:**
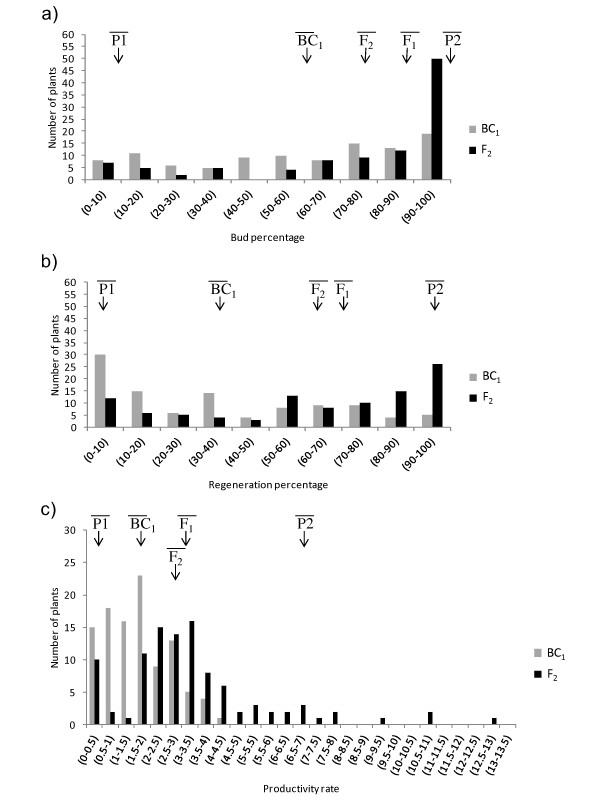
**Population distributions for regeneration traits**. a) The percentage of explants with buds (B), b) The percentage of explants with plants (R) and c) The percentage of plants per explant with shoots (PR). The F_2 _population (dark) is derived from selfing an F_1_, the result of a cross between the tomato cv. Anl27 (P1) and *S. pennellii *PE-47 (P2). The BC_1 _population (grey) is the result of crossing the tomato cv. Anl27 and the F_1 _plants. Maternal (P1), Paternal (P2), F_1_, F_2 _and BC_1 _mean values are indicated by arrows.

The F_2 _and BC_1 _populations were evaluated for regeneration using explants from the parents and F_1 _plants as controls (Table 1). The phenotypes are shown in Additional File 1. The distribution obtained for each individual trait as well as the means for controls in this assay are presented in Figure 1. Mean values for B, R and PR in the F_2 _population are between F_1 _and tomato (P1), but skewed towards F_1_. For the PR trait, some F_2 _plants were in a range higher than the *S. pennellii *parent (P2). This can be considered transgressive segregation. BC_1 _yielded mean values for B, R and PR that were intermediate between F_1 _and cv. Anl27 (Figure 1).

B and R show a high correlation (r = 0.88/0.79 p < 0, 001 for F_2 _and BC_1 _data, respectively), which suggests common or linked genes controlling these traits. The correlation between PR and both B and R was lower (r = 0.56/0.52 p < 0, 001; 0.66/0.66 p < 0, 001 for PR and B and R for F_2 _and BC_1_, respectively) indicating that different genes may influence the PR trait and/or variations between different biological samples are higher in PR.

### Linkage maps

**Figure 2:**
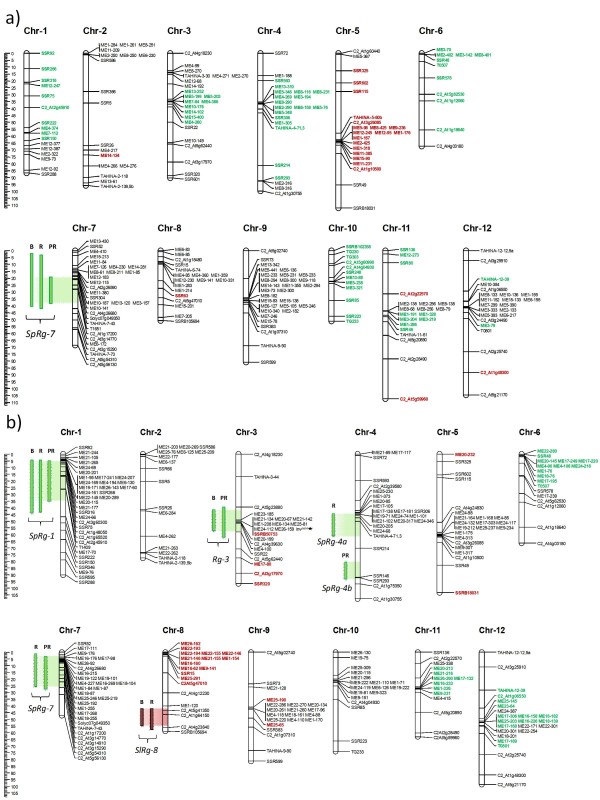
**a) Tomato genetic linkage map of F_2 _population derived from *S. lycopersicum *(cv. Anl27) × *S. pennellii *(PE-47) and QTLs detected for regeneration traits by IM**. b) Tomato genetic linkage map of BC_1 _population derived from *S. lycopersicum *(cv. Anl27) × F_1 _(cv. Anl27 × PE-47) and QTLs detected for regeneration traits by rMQM. The segregated data were classified into 12 linkage groups, which corresponded to the Tomato-EXPEN 2000 map; italics indicate markers with segregation significantly skewed (P < 0.05) in favour of parent alleles. The colors specify the direction of the segregation distortion (red: markers skewed toward the alleles of cultivated tomato; green: markers skewed toward the alleles of the wild parent). Green bars reflect QTLs from S. *pennellii*: *SpRg-1, Rg-3, SpRg-4a, SpRg-4b *and *SpRg-7*; the red bar reflects the *SlRg-8 *QTL from S. *lycopersicum*. Regeneration traits: B (Bud percentage), R (Regeneration percentage) and PR (Productivity rate). The black star labels the acid invertase gene (inv*^penn^*) mapped on chromosome 3 included in the *Rg-3 *QTL range.

Genetic linkage maps were constructed from 106 F_2 _and 113 BC_1 _plants genotyped with SSR, COSI, COSII, CAPS and AFLP markers (Figure 2). Of the 149 SSR and 97 other markers (86 COSII, 6 COSI, 5 CAPS) assayed, 78 SSR and 59 (51 COSII, 4 COSI, 4 CAPS) markers exhibited codominant polymorphisms. These markers were obtained from the Sol Genomics Network (SGN) webpage at http://www.sgn.cornell.edu/with the exception of 60 SSRs that were designed following the procedure described in Materials and Methods (see Additional File 2).

For the F_2 _linkage map (Figure 2a), a total of 246 polymorphic loci were used, including 151 AFLP, 53 SSR, 35 COSII, 3 COSI and 4 CAPS markers. The markers were aligned in 12 linkage groups, with LOD scores ≥ 3.0. The average number of markers per linkage group was 20 and markers were well distributed over all the 12 linkage groups. The F_2 _map spans 963.85 cM with an average interval of 3.72 cM between adjacent markers. There were five intervals > 25 cM in chromosomes 2, 4, 5 and 11. A total of 268 polymorphic loci were used to assemble the genetic linkage map of BC_1 _(Figure 2b), including 174 AFLP, 46 SSR, 43 COSII, 3 COSI and 2 CAPS markers. The markers were distributed over 12 linkage groups with LOD scores ≥ 3.0. The average number of markers per linkage group was 22. The total genetic distance covered by the markers was 1014.94 cM, with an average interval of 4.12 cM between adjacent markers. The markers were well distributed over all the 12 linkage groups with only two intervals ≥ 25 cM in chromosomes 5 and 10. Marker distribution in both maps indicates that they will be useful for tagging the traits studied.

The order and placement of SSR markers were in agreement with the *S. lycopersicum *x *S. pennellii *reference tomato-EXPEN 2000 map (SGN) with the exception of TAHINA-6-64 (*in silico *designed), which was expected to be positioned on chromosome 6 (position 64) but is positioned on chromosome 8 (position 8.85) in our F_2 _map.

### Distorted segregation

42.45% of the mapped markers deviated significantly from the expected 1:2:1 segregation ratio for the F_2 _generation at P < 0.05 (Figure 2a). Segregation distorted markers (SDMs) were mainly observed on chromosomes 1 (0.00-63.17 cM), 3 (33.24-38.85 cM), 4 (19.74-92.09 cM), 5 (12.60-72.26 cM), 6 (0.00-55.38 cM) and 10 (0.00-51.24 cM). SDMs were generally caused by a surplus of *S. pennellii *homozygotes, with the exception of that observed on chromosome 5.

In the BC_1 _population (Figure 2b), SDMs were fewer (30.3%) than in F_2_, and were observed mainly on chromosomes 6 (0.00-6.75 cM), 8 (0.00-15.40 cM), 11 (25.80-27.48 cM) and 12 (28.23-60.63 cM). The distortion on chromosome 8 was caused by a surplus of tomato homozygotes, whereas distortions on the other chromosomes were caused by an excess of hybrid genotypes.

### QTL Identification

In order to identify QTLs, we first used Interval Mapping (IM) analysis that resulted in the identification of one QTL in the F_2 _population and six in the BC_1 _population (See Additional Files 3, 4, 5 and 6). The QTL identified in F2, located on chromosome 7, overlapped for the three traits. In the BC_1 _analysis, this QTL also appeared for the R and PR traits. However, in this population, another five QTLs were identified on chromosomes 1, 3, 4 (at two different areas: 4a and 4b) and 8. All these QTLs were confirmed by restricted Multiple QTL Mapping (rMQM) analysis (Figure 2b, Table 2). With the exception of the QTL on chromosome 8, all QTLs come from *S. pennellii*. These QTLs were named by their origin, *Sp *for *S. pennellii *or *Sl *for *S. lycopersicum*, followed by *Rg *(referring to regeneration) and the number of the chromosome on which they were located.

**Table 2:** QTLs for shoot regeneration traits (Bud percentage (B), Regeneration percentage (R) and Productivity Rate (PR)) found to be significant at the empirical genome wide mapping threshold by restricted Multiple QTL Mapping (rMQM) in BC_1 _and Interval Mapping (IM) in F_2_

Test	QTL analysis	Trait	QTL	Genome wide significant threshold level (P < 0.05)	Chr	Start (cM)	Finish (cM)	Coverage (cM)	LOD Peak	Position of LOD peak (cM)	Peak marker ^a^	%variance explained	Estimated additive effect	Estimated dominance effect
BC_1_	rMQM	B	*SpRg-1*	2.7	1	3.87	44.42	40.55	7.12	22.47	C2_At1g65520/C2_At2g45910	23.9	-31.56	
BC_1_	rMQM	R	*SpRg-1*	2.7	1	3.87	43.42	39.55	5.52	24.47	C2_At1g65520/C2_At2g45910	15.0	-24.10	
BC_1_	rMQM	PR	*SpRg-1*	2.8	1	3.87	34.42	30.55	4.19	22.47	C2_At1g65520/C2_At2g45910	10.2	-0.70	

BC_1_	rMQM	B	*Rg-3*	2.7	3	42.41	55.80	13.39	4.64	50.47	ME20-199	12.2	-21.60	
BC_1_	rMQM	PR	*Rg-3*	2.8	3	32.77	63.10	30.33	4.26	50.47	ME20-199	10.6	-0.68	

BC_1_	rMQM	R	*SpRg-4a*	2.7	4	44.39	61.24	16.85	4.94	50.24	TAHINA-4-71, 3	13.3	-22.29	

BC_1_	rMQM	PR	*SpRg-4b*	2.8	4	81.33	93.18	11.85	3.08	86.33	SSR214/SSR146	7.4	-0.63	
F_2_	IM	B	*SpRg-7*	3.7	7	2.20	40.28	38.08	6.84	19.51	ME10-141/C2_At4g26680	27.0	-22.20	12.32
F_2_	IM	R	*SpRg-7*	3.6	7	4.50	40.28	35.78	6.18	19.51	ME10-141/C2_At4g26680	24.8	-23.29	13.63
F_2_	IM	PR	*SpRg-7*	4.4	7	19.51	36.28	16.77	5.72	28.28	C2_At1g17200	23.1	-1.53	-0.55
BC_1_	rMQM	R	*SpRg-7*	2.7	7	0.00	25.08	25.08	5.47	13.44	TAHINA-7-43	14.9	-23.13	
BC_1_	rMQM	PR	*SpRg-7*	2.8	7	3.54	28.23	24.69	5.28	13.44	TAHINA-7-43	13.5	-0.77	

BC_1_	rMQM	B	*SpRg-8*	2.7	8	41.18	53.37	12.19	3.84	46.37	C2_At1g64150	12.2	21.25	
BC_1_	rMQM	R	*SpRg-8*	2.7	8	42.18	58.90	16.72	4.25	53.37	C2_At1g64150/C2_At4g23840	9.3	19.35	

^a^In case of the absence of a peak marker, loci flanking the likely peak of a QTL are shown.

### QTLs for regeneration traits in the BC_1 _population

#### Bud percentage (B)

IM analysis identified two QTLs on chromosomes 1 and 8 (*SpRg-1 *and *SlRg-8*; Additional File 4). *SpRg-1 *has a maximum LOD score of 5.87 and is spanned by markers SSR316 and ME17-70. This QTL explained 22.9% of the phenotypic variation of the B trait. *SpRg-8*, with a maximum LOD score of 2.8, including just the C2_At1g64150 marker, explained 11.7% of the phenotypic variation in B. rMQM analysis, using C2_At2g45910 (chromosome 1) and C2_At1g64150 (chromosome 8) markers as cofactors, confirmed those QTLs detected by IM and detected a new one on chromosome 3 (Figure 2b, Additional File 4). QTL characteristics are shown in Table 2. Collectively, these QTLs explained 34.6% and 48.3% of phenotypic variance in IM and rMQM, respectively.

#### Regeneration percentage (R)

IM analysis identified three QTLs located on chromosomes 1, 4 and 7 denominated *SpRg-1, SpRg-4a *and *SpRg-7*, respectively. The three QTLs had maximum LOD scores of 4.20, 3.92 and 3.86, and each explained around 16-17% of the phenotypic variation (see Additional File 5). rMQM analysis, using C2_At2g45910 (chromosome 1), TAHINA-4-71.3 (chromosome 4) and TAHINA-7-43 (chromosome 7) markers as cofactors, confirmed all QTLs detected by IM and detected the *SlRg-8 *QTL (Figure 2b, Additional File 5, Table 2). In this case, the percentage of the phenotypic variation explained by each QTL was 15% for *SpRg-1*, 13.3% for *SpRg-4a*, 14.9% for *SpRg-7 *and 9.3% for *SlRg-8*. Collectively, these QTLs explained 48.7% and 52.5% of the phenotypic variance in IM and rMQM, respectively.

#### Productivity rate (PR)

IM detected the QTLs located previously for B and R on chromosomes 1, 3 and 7 (Figure 2b, Additional File 6), as well as another QTL on chromosome 4, denominated *SpRg-4b*. The maximum phenotypic variation for PR (17.4%) is explained by *SpRg-7*, and the lowest (11.9%) by a QTL on chromosome 3. rMQM analysis, using SSR92 (chromosome 1), ME20-199 (chromosome 3), SSR146 (chromosome 4) and TAHINA-7-43 (chromosome 7) markers as cofactors, confirmed the QTLs detected by IM (Table 2).

### Mapping tomato candidate genes

We selected the acid invertase gene linked to the *Rg-2 *regeneration gene of *S. chilense *[22] and the *LESK1 *gene, described as a marker in tomato for *in vitro *regeneration competence [27], as the tomato candidate genes.

The amplification products of the acid invertase gene marker (*inv^penn^*) produce fragments of different sizes: 162 bp for *S. lycopersicum *cv. Anl27 and 173 bp for *S. pennellii *(see Additional File 7). Thus, inv*^penn ^*was used for mapping the BC_1 _population (Figure 2b, Additional Files 4 and 6). It was located in the QTL detected on chromosome 3, between the C2_At5g23880 and SSRB50753 markers, at positions 49.9 cM and 49.93 cM, respectively. For this reason, we named this QTL *Rg-3 *(a putative allele of *Rg-2*).

The *LESK1 *gene is located in the SGN Tomato-EXPEN 2000 map on chromosome 3 between markers C2_At4g18230 and cLPT-5-e7 (7 - 15 cM). As a result, in our BC_1 _map, *LESK1 *must be placed between C2_At4g18230 and TAHINA-3-44 (7 - 44 cM). Thus, this candidate gene is outside the located *Rg-3 *QTL.

### Exploring QTLs

The official annotation for the tomato genome provided by the International Tomato Annotation Group at the SGN was used to carry out a preliminary search for related regeneration genes near the identified QTL peaks. We found a histidine kinase in *SpRg-7*, several serine/threonine kinases in all identified QTLs, ethylene response factors (ERFs) in all identified QTLs with the exception of *SpRg-4b*, cyclines in *SpRg-1, Rg-3, SpRg-4a *and *SpRg-7 *and MADS-box in *SpRg-1, SpRg-4a *and *SpRg-7*.

## Discussion

The wild tomato species *S. peruvianum, S. pimpinellifolium *L. *and S. chilense *were used as sources of regeneration genes in order to study the genetics of the *in vitro *regeneration in tomato [7,9,21]. In this study, we used one accession of *S. pennellii *(PE-47) as the high regeneration parent [29]. This accession, along with a previously selected low regenerating tomato cultivar (cv. Anl27), was used to develop two mapping populations (F_2 _and BC_1_). The use of the introgression lines of *S. pennellii *in the M82 tomato background [30] had been previously ruled out for this analysis because of the high regeneration ability of both parent lines (data not shown). Data in Figure 1 and Table 1 seem to indicate complete dominance for B, partial dominance for R and additive effects for PR. This is in agreement with other reported studies on tomato where dominance, to different degrees, depending on the regeneration trait studied, was also reported [21,22,24,25]. B and R traits show a high correlation in both populations, suggesting that common or linked genes control these traits. The correlation between PR and both B and R was lower. This could imply that other genes may be influencing the PR trait and/or variations between different biological samples are higher in PR (for instance, competition for development due to the presence of different shoots in a similar explant area). Thus, the low sample size may be also a possible explanation for the lower correlation.

Some descendants in the F_2 _population showed phenotypes for the PR trait that are more extreme that those shown by the regenerating parent line (Figure 1). Transgressive segregation has already been described in other reports in relation to the genetic control of plant regeneration [31-33], and suggests poligenic inheritance [34]. It also suggests the existence of alleles that promote, and others that inhibit, *in vitro *regeneration, with only some of the alleles with positive effects occurring in the same parent [34]. In fact, in this study, the *SlRg-8 *QTL that contributes to regeneration came from the low regenerating parent.

Plant regeneration from cultured tissues is assumed to fall under quantitative genetics [34], although evidence in tomato [22,25] and other vegetables [35-37] indicates that just a few genes could be responsible for regeneration. We identified 6 QTLs in the BC_1 _analysis, which is indicative of the participation of a large number of genes in this character. These QTLs are situated on chromosomes 1, 3, 4, 7 and 8 (Figure 2b). The percentage of variance explained by each QTL ranges from 7.4 to 27%, which is in accordance with the most common range (6-26%) reported in the genetic mapping of QTLs for tissue culture response in plants [34]. We used three traits (B, R and PR) as a measurement of regeneration capability that could be useful for detecting chromosome regions that act at different times.

In the F_2 _population, only the QTL of chromosome 7 was identified for all analyzed traits (Additional File 3); the SDMs observed in most chromosome areas where QTLs were detected in the BC_1 _population are most likely the cause (Figure 2). The SDMs on chromosomes 1, 3, 6, 10 and 11 were also observed in similar areas in the Tomato-EXPEN 2000 map [38]. SDMs affect the detection power of QTLs when QTLs and SDMs are closely linked [39], as occurred in our case. Deviation from the expected segregation ratio is a common feature of inter-specific tomato crosses [40]. To wit: in a F_2 _population from *S. lycopersicum *x *S. pennellii*, De Vicente and Tanskley [41] reported a skewness rate of up to 80%.

In the BC1 population, three QTLs were detected for B: *SpRg-1, SpRg-3 *and *SlRg-8*. These QTLs may be associated with the first stages of regeneration, that is, hormonal induction response and bud formation. *SpRg-1*, which explained the highest percentage of variation for B (23.9%), was also identified for the R and PR traits. Given that bud formation is a necessary prerequisite for the production of shoots, it was expected that this major QTL for B would be found for R and PR, which in fact turned out to be the case (Table 2). For R and PR, a common QTL on chromosome 7 (*SpRg-7*) was also identified. In addition, two QTLs were detected for R (*SpRg-4a *and *SlRg-8*) and PR (*SpRg-4b *and *Rg-3*). All these QTLs seem to be involved in the development of buds into shoots. As can also be observed in this study, common QTLs for the different regeneration traits, as well as a higher number of QTLs for traits related to plant development compared to those associated with bud induction, have been reported in different studies [42,43]. For instance, in Arabidopsis, Schianterelli et al. [43] found a common area of chromosome 1 in all analyzed parameters, a peak in chromosome 4 and another in chromosome 5 when they analyzed the total number of regenerated shoots. In wheat, Ben Amer et al. [42] identified three QTLs, two that affect green spot initiation and shoot regeneration and a third that only influences plant formation.

A partial common genetic system controlling the regeneration frequency of diverse types of explants has been reported by Molina and Nuez [36] in melon. This indicates that using different explants for loci detection may lead to the identification of some common QTLs, but also to the possible identification of other new QTLs. Root explants were used by Koornneef et al. [25] and Satoh et al. [22] for phenotyping, at which point two alleles for regeneration ability were located on chromosome 3 of tomato. In the present study, leaves were used for phenotyping and a QTL (*Rg-3*) in a similar area of chromosome 3 was detected in addition to other QTLs that influence regeneration and were identified on chromosomes 1, 4, 7 and 8. Differences in root and leaf explants for QTL identification were also found in *Arabidopsis thaliana *[43].

Koornneef et al. [25] located a dominant allele from *S. peruvianum *(*Rg-1*) near the middle of chromosome 3 that determines efficient shoot regeneration in tomato root explants. Satoh et al. [22] mapped a putative allele (*Rg-2*) from *S. chilense *on this chromosome. The acid invertase gene, reported as a marker linked to *Rg-2*, was chosen for mapping *Rg-2 *in our population derived from *S. pennellii*. The polymorphisms detected in our parents allow us to map this gene in the QTL detected on chromosome 3 that we named *Rg-3*. We consider *Rg-3 *to be a putative allele of the *Rg-2 *gene. Allelism must be confirmed.

The other gene chosen as a candidate was *LESK1*, which encodes a serine/threonine kinase, and was reported as a marker of competence for *in vitro *regeneration in tomato [27,28]. This gene was positioned on chromosome 3, but it is not located in the *Rg-3 *QTL.

The recent release of the entire genome sequence of tomato provides a powerful tool for interrogating QTL data. In this respect, we have taken a preliminary look at genes located at the peak areas of the detected QTLs, and which could be related to organogenesis. Histidine kinases were reported as cytokinin receptors [15-17]. In our QTL peaks, only one histidine kinase is located in the *SpRg*-7 QTL. The candidate tomato gene, *LESK1*, which has been described as a marker for *in vitro *competence, encodes a serine/threonine kinase. We looked for serine/threonine kinases and found this kind of protein in all identified QTLs. Other putative candidate genes could be *ESR1 *and its paralogue, *ESR2*, from Arabidopsis, which are the best-characterized genes related to regeneration [18,19]. These genes code for ethylene response factors (ERF). We found ERFs, which contain the AP2 domain, in all analysed QTLs with the exception of *SpRg-4b*. Cyclines related to cell division [13] were found in *SpRg-1, Rg-3, SpRg-4a *and *SpRg-7*. MADS-box genes, which have been correlated to adventitious regeneration induction and regulation [44,45], were found in the *SpRg-1, SpRg-4a *and *SpRg-7 *QTL peaks.

## Conclusions

The results obtained in this study may very well represent a significant step toward the goal of understanding the processes underlying tomato tissue culture and regeneration responses. We have situated six QTLs on chromosomes 1, 3, 4, 7 and 8, five from *S. pennellii *and one from *S. lycopersicum*. The most important QTLs are *SpRg-1*, which is most likely associated with the morphogenetic response, and *SpRg-7*, which promotes bud development. A QTL detected on chromosome 3, *Rg-3*, likely contains a putative allele of the *Rg-1 *and *Rg-2 *genes, as is shown by mapping the acid invertase gene linked to *Rg-2*. QTLs detected on chromosomes 8 and 4 most likely contain genes influencing bud formation and development, respectively.

## Methods

### Plant materials and growing conditions

*S. pennellii *PE-47, which showed a high ability for regeneration [29], and the tomato cultivar Anl27 (cv. Anl27), with a low ability for regeneration, were chosen for obtaining the mapping population. The initial genotypes were established *in vitro*, starting with the sterilization of seeds by immersion for 10 min in a solution of 25% commercial bleach (40 g L^-1 ^active chlorine), being then washed twice with sterile deionized water for 5 min each and then sown in Petri dishes containing nutrient medium (Murashige and Skoog [46] salts including vitamins, 2% sucrose, 0.6% plant agar (DUCHEFA, the Netherlands). The pHs of the media were adjusted to 5.8 before sterilization at 121°C for 20 min. Cultures were incubated in a growth chamber at 26°C ± 2°C under a 16h photoperiod with cool white light provided by Sylvania cool white F37T8/CW fluorescent lamps (90 μmol m^-2 ^s^-1^). Clones of one plant of each genotype were obtained and maintained in *in vitro *culture. The clones were multiplied by transferring nodes to tubes with fresh basal medium (BM: Murashige and Skoog -[46]- salts including vitamins, 1.5% sucrose and 7 g L^-1 ^plant agar) every 3-4 weeks. The tubes were 15 cm in length and 22 mm in diameter, with 15 ml of medium per tube.

### Mapping population

One clone of tomato and another of *S. pennellii *were transferred to a greenhouse in order to obtain the F_1 _plant that was reintroduced *in vitro *by disinfection of shoots following a similar procedure as that carried out for seed sterilization. F_2 _and BC_1 _populations were obtained and seeds were germinated *in vitro *as described above.

The F_2 _mapping population was composed of 106 individuals obtained from selfing one F_1 _plant, the result of a cross between the tomato cv. Anl27 (P1) and *S. pennellii *PE-47 (P2). The backcross (BC_1_) mapping population, composed of 113 plants, was obtained by crossing the cv. Anl27 and the F_1 _plant. To allow the test to be reproduced, the F_1 _plant and F_2 _and BC_1 _individuals were clonally replicated and maintained *in vitro *as described above.

### Evaluation of the regeneration capacity

A first assay was performed with cloned P1, P2 and F_1 _plants. Leaf disks (0.6-0.8 cm^2^) obtained from *in vitro *cultured plants that were at a similar growing stage were placed with the abaxial side in contact with the shoot induction medium (SIM) containing Murashige and Skoog salts [46], 3% sucrose, 7% plant agar and 0.2 mg L^-1 ^zeatin riboside (ZR). This growth regulator was sterilized by filtration and added to the sterile SIM. After 30 days of culture on SIM, the explants were transferred to BM for 20 days. In this medium, buds develop into shoots. For each genotype, five explants per plate (90 × 25 mm with 40 ml of medium per plate) and 10 repetitions per genotype were evaluated. At the end of the experiment, the following variables were analyzed:

-Bud percentage (B): number of explants with buds × 100/total number of cultured explants.

-Regeneration percentage (R): number of cultures that differentiated into completely developed shoots × 100/total number of cultured explants.

-Productivity rate (PR): total number of completely developed shoots/total number of cultured explants that regenerated plants.

In a second assay, leaf explants of F_2_, BC_1_, P1, P2 and F_1 _plants were tested as explained above. In this case, for each genotype, five explants per plate and 4 repetitions per genotype were evaluated. Data for regeneration was obtained for 102 genotypes of the F_2 _population and 104 genotypes of BC_1_. The average value for each trait and genotype was used for QTL analysis.

To assess the effect of genotype on regeneration ability, data from the genetically uniform classes (P1, P2 and F_1_) were subjected to a unifactorial analysis of variance (ANOVA), and then means for the different traits were separated by a Duncan test. The correlations between the different traits were calculated using the Statgraphics Plus 4.0 software.

### Genotyping

#### Preparation of genomic DNA

Young leaves from *in vitro*-cultured plants were collected and immediately frozen with liquid nitrogen and then stored at -80°C. DNA was prepared based on the modified CTAB method of Doyle and Doyle [47]. Subsequently, quality and quantity of the DNA was evaluated on 0.8% agarose gel stained with ethidium bromide and using the NanoDrop^® ^ND-1000 Spectrophotometer.

#### Amplified fragment length polymorphism (AFLP) procedure

AFLPs were obtained following de Vos et al. [48] procedure. Fifteen and sixteen selective combinations of primers were used for the F_2 _and BC_1 _populations, respectively. The code of each selective combination is specified in Table 3. Each code followed by the number corresponding to each obtained band (size in bp) is used to name the polymorphic AFLPs. Electrophoresis of the PCR products was conducted using an ABI PRISM 310 Genetic Analyzer (PerkinElmer Applied Biosystems, Foster City, California, USA). GeneScan™ 600 LIZ^® ^Size Standard, with fluorophore LIZ, was used as a molecular size marker. Raw data were analyzed with the GeneScan 3.1.2 analysis software (PerkinElmer Applied Biosystems) and the resulting GeneScan trace files were imported into Genographer 1.6.0. The AFLP fragments between 60 to 380 bp were scored in Genographer as present (1) or absent (0).

**Table 3:** Selective combinations of primers used for F_2 _and BC_1 _genotyping

Code	Mapping population	Selective primers combination
ME1	F_2_, BC_1_	*MseI *CTA*-EcoRI *AAC
ME2	F_2_	*MseI *CAA*-EcoRI *ACC
ME3	F_2_	*MseI *CAA*-EcoRI *ACG
ME4	F_2_, BC_1_	*MseI *CAA*-EcoRI *AGC
ME5	F_2_	*MseI *CAC*-EcoRI *ACA
ME6	F_2_, BC_1_	*MseI *CAC-*EcoRI *ACG
ME7	F_2_	*MseI *CAC*-EcoRI *AGC
ME8	F_2_, BC_1_	*MseI *CAA*-EcoRI *ACA
ME9	F_2_, BC_1_	*MseI *CAA*-EcoRI *AAC
ME10	F_2_	*MseI *CTA*-EcoRI *AGC
ME11	F_2_	*MseI *CTC*-EcoRI *AGC
ME12	F_2_	*MseI *CCG*-EcoRI *AAC
ME13	F_2_	*MseI *CCG*-EcoRI *ACC
ME14	F_2_	*MseI *CCG*-EcoRI *ACG
ME15	F_2_	*MseI *CTC*-EcoRI *AGG
ME16	BC_1_	*MseI *CAA*-EcoRI *ACT
ME17	BC_1_	*MseI *CTA*-EcoRI *ACC
ME18	BC_1_	*MseI *CTA*-EcoRI *ATG
ME19	BC_1_	*MseI *CTA*-EcoRI *ACA
ME20	BC_1_	*MseI *CCT*-EcoRI *ACC
ME21	BC_1_	*MseI *CCT*-EcoRI *AAC
ME22	BC_1_	*MseI *CCT*-EcoRI *ATG
ME23	BC_1_	*MseI *CCT*-EcoRI *ACA
ME24	BC_1_	*MseI *CAC*-EcoRI *ACC
ME25	BC_1_	*MseI *CAC*-EcoRI *ATG
ME26	BC_1_	*MseI *CAC*-EcoRI *AGG

#### Microsatellites (SSRs)

One hundred and forty-nine SSR markers were used to detect polymorphism between P1 and P2, which included 89 SSRs previously reported and mapped onto the Tomato-EXPEN 2000 available at SGN [49,50], along with 60 new SSRs: 18 from the COMAV research group "Aprovechamiento de la variabilidad estraespecífica en la mejora del tomate" and 42 designed from sequences deposited in Genbank (see Additional File 2). Primer pairs were designed from these sequences using the SSR Primer 3 tool http://frodo.wi.mit.edu/[51]. The criteria used for designing the primers were as follows: the primer Tm ranged from 55 to 65°C and GC content was 50%. The presence of G or C bases within the last five bases from the 3' end of primers (GC clamp), which helps promote specific binding at the 3' end, was taken into account. In order to design the SSRs, wherever possible the AT/TA repetitions were selected based on the results obtained by Frary et al. [49].

All the SSRs, with the exception of those specified below, were labelled following the M13-tail method described by Schuelke et al. [52]. DNA amplification was carried out in volumes of 15 μL using a sample of 10 ng of DNA. The reaction mixture contained 1.5 μL 10 × PCR buffer [75 mM Tris-HCl (Ph 9.0), 50 mM KCl, 20 mM (NH_4_)_2_SO_4 _and 0.001% BSA], 2 mM MgCl_2_, 200 μM dNTPs, 0.133 μM of primers, 0.2 μM of fluorescent labelled M13 primer and 0.3 units of *Taq*I DNA polymerase (Need S. L., Valencia, Spain). An Eppendorf 5333 Thermal Cycler was used. The PCR parameters included the following: an initial 3 min at 94°C; 35 cycles, each with 30 s DNA denaturation at 94°C; 45 s at an annealing temperature (depending on the primer combination Tm) and a 1 min extension at 72°C, and a final extension of 10 min at 72°C. Amplified bands were visualized using a LI-COR sequencing gel (DNA LI-COR 4300; LI-COR Biosciences, Lincoln, Nebraska, USA); 10 μl of loading buffer (95% formamide, 2 mM EDTA, 0.001% bromophenol blue) and 5 μl of deionized water were added to the 5 μl PCR mix (2.5 μl of each IRDye 700 or IRDye800-labeled) samples which were denatured at 96°C for 8 min. Electrophoresis was performed in denaturing conditions at 50°C, using 6% acrylamide gels in TBE buffer.

The SSR356, SSR73, SSR248, SSR46 markers in which polymorphisms were visible in the agarose gels were amplified in volumes of 23.32 μl with: 10 ng of DNA, 1.6 mM MgCl_2_, 171.52 μM dNTPs, 0.214 μM of primers, 2.5 μl of 10 × PCR buffer, and 0.6 U *Taq*I DNA polymerase. The PCR conditions were similar to those applied before, with the exception of a final extension of 30 min in this case. Amplified bands were run in standard agarose gels (1 or 2%) in TAE buffer at 100V and visualized by ethidium bromide staining.

#### Conserved ortholog set (COS) and cleaved amplified polymorphic sequence (CAPS) markers

Ninety-six markers (86 COSII, 6 COSI, 4 CAPS) from the Tomato-EXPEN 2000 map [53,54] and one developed CAPS marker (Solyc07g049350) were tested for polymorphism between the P1 and P2 parents. The restriction enzymes used when required were those indicated in the SGN database. When restriction enzymes was needed, the protocol described in the commercial product's instructions (Fermentas, York, UK or Biolabs, Takara, Japan) was followed.

The PCR reaction was performed in a total volume of 12 μL using a sample of 10 ng of DNA. The reaction mixture contained 1.5 mM MgCl_2_, 200 μM dNTPs, 0.25 μm of primers, 1.2 μL PCR buffer 10X, and 0.3 U *Taq*I DNA polymerase. Amplification was performed using an Eppendorf 5333 Thermal Cycler, which was programmed as follows: 5 min at 94°C, 35 30-s cycles each at 94°C, 1 min at Ta (depending on the primer combination Tm) and a 2 min extension at 72°C, with a final stage of 10 min at 72°C. Amplified bands were separated by 1 or 2% agarose electrophoresis in TAE buffer at 100V, and visualized by ethidium bromide staining.

### Map construction and QTL mapping

Linkage analysis for both mapping populations was performed with the JoinMap^® ^4.0 software [55]. Markers were grouped into linkage groups at LOD ≥ 3, with the exception of those in chromosomes 9 and 10 of the BC_1 _mapping population with LOD ≥ 2. Order was determined with a recombination threshold of 0.40 and distances were calculated using the Kosambi mapping function (Kosambi 1944). For the genetic map construction, AFLP, SSR, COS and CAPS markers were used (Additional File 8). The segregation ratio of alleles was evaluated for each locus by the Chi-square test with a significance threshold of P < 0.05. The expected segregation ratios were 3:1 and 1:1 for F_2 _and BC_1_, respectively. Visual representations of the marker maps were created with the MapChart software [56].

QTL analysis on the F_2 _and BC_1 _phenotypic data sets was performed with the MapQTL^® ^6.0 software [57]. Significance thresholds for the LOD values, corresponding to a genome-wide false discovery rate of 5% (p < 0.05) were calculated by genome-wide permutation tests using 1, 000 permutations. Firstly, IM analysis was performed (simple Interval Mapping). Then, if many putative QTLs were detected by IM, markers close to the likelihood peaks of the detected QTLs were used as cofactors for rMQM (also called composite Interval Mapping) analyses.

### Locating candidate genes and looking for other regeneration-related genes

The *S. pennellii *acid invertase gene (inv*^penn^*) was analyzed and mapped as a marker. Primers described by Harada et al. [58] were used for DNA amplification using conditions previously described for COS and CAPS markers. Amplified bands were separated using the multicapillary electrophoresis QIAxcel System (Qiagen, Valencia, California, USA). We searched for the location of the *LESK1 *gene at SGN and for its nearest markers at International Tomato Annotation Group. This database was also used for looking for genes putatively related to organogenesis.

## Authors' contributions

CG obtained the mapping populations. CT conducted the population phenotyping and genotyping and participated in the drafting. CT and SV performed the map construction and QTL mapping. CG collaborated in the phenotyping and genotyping and conceived, supervised and drafted the manuscript. FN conceived of the study and contributed to critically reviewing the manuscript. All authors read and approved the final manuscript.

## Supplementary Material

Additional file 1 **Regeneration response of leaf explants**. Regeneration response of leaf explants from parents [tomato (cv. Anl27); *S. pennellii *(PE-47)], F_1_, F_2 _and BC_1 _populations, cultured on shoot induction medium (SIM) for 30 days and transferred to basal medium (BM) for 20 days. Click here for file

Additional file 2 **In silico-designed SSR markers**. Table with the name, band size, repeat motif, temperature of annealing and primers sequences of in silico-designed SSR markers. Click here for file

Additional file 3 **Genetic location and LOD score profile of the F_2_-QTLs for regeneration components detected by Interval Mapping on chromosome 7 (*SpRg-7*)**. Genetic location and LOD score profile of the F_2_-QTLs for regeneration components (Bud percentage (B), Regeneration percentage (R) and Productivity Rate (PR)). On the left, projections of QTLs as black bars indicate the *SpRg-7 *for B, R and PR traits. The vertical dotted line indicates the 95% significant threshold value for declaring a QTL (B LOD threshold = 3.7) (R LOD threshold = 3.6) (PR LOD threshold = 4.4). Map position (cM) and distances are based on the genetic linkage map developed in this study. QTLs characteristics in attached table. Click here for file

Additional file 4 **Genetic location and LOD score profile of the BC_1_-QTLs for Bud percentage (B), detected on chromosomes 1 (*SpRg-1*), 3 (*SpRg-3*) and 8 (*SlRg-8*)**. Results from the Interval Mapping (IM) and restricted Multiple QTL Mapping (rMQM) approaches. On the left, projections as black bars (IM) and grey bars (rMQM) indicate the range of *SpRg-1, SpRg-3 *and *SlRg-8 *QTLs for B. The vertical dotted line indicates the 95% significant threshold value for declaring a QTL (B LOD threshold = 2.7). The horizontal dotted line indicates the position of the acid invertase gene (inv*^penn^*) marker included in the chromosome 3 QTL range. Map position (cM) and distances are based on the genetic linkage map developed in this study. Click here for file

Additional file 5 **Genetic location and LOD score profile of the BC_1_-QTLs for Regeneration percentage (R), detected in this study on chromosomes 1 (*SpRg-1*), 4 (*SpRg-4a*), 7 (*SpRg-7*) and 8 (*SlRg-8*)**. Results from the Interval Mapping (IM) and restricted Multiple QTL Mapping (rMQM) approaches. On the left, projections as black bars (IM) and grey bars (rMQM) indicate the range of *SpRg-1, SpRg-4a, SpRg-7 *and *SlRg-8 *QTLs for R. The vertical dotted line indicates the 95% significant threshold value for declaring a QTL (R LOD threshold = 2.7). Map position (cM) and distances are based on the genetic linkage map developed in this study. Click here for file

Additional file 6 **Genetic location and LOD score profile of the BC_1_-QTLs for Productivity Rate (PR), detected in this study on chromosomes 1 (*SpRg-1*), 3 (*SpRg-3*), 4 (*SpRg-4b*) and 7 (*SpRg-7*)**. Results from the Interval Mapping (IM) and restricted Multiple QTL Mapping (rMQM) approaches. On the left, projections as black bars (IM) and grey bars (rMQM) indicate the range of *SpRg-1, SpRg-3, SpRg-4b *and *SpRg-7 *for PR. The vertical dotted line indicates the 95% significant threshold value for declaring a QTL (PR LOD threshold = 2.8). Horizontal dotted lines indicate the position of the acid invertase gene (inv*^penn^*) marker included in the chromosome 3 QTL range. Map position (cM) and distances are based on the genetic linkage map developed in this study. Click here for file

Additional file 7 **Polymorphic acid invertase gene marker (*inv^penn^*)**. Amplified bands separated using the multicapillary electrophoresis QIAxcel System. Lane 1: *S. lycopersicum *L. (Anl27), band size (~162bp). Lane 2: *S. pennellii *PE-47, band size (~173bp). Lane 3: F_1 _Hybrid *S. lycopersicum *L. (Anl27) × *S. pennellii *PE-47, both bands (~162bp-~173bp). Lane 4: negative control. Click here for file

Additional file 8 **Markers used for genotyping the F_2 _and BC_1 _population**. SSR, COS, COSII, CAP markers used for genotyping the F_2 _and BC_1 _population. Click here for file
