# Supplementary figures and images for: Localization of QTLs for in vitro plant regeneration in tomato
Source: BMC Plant Biol. 2011 Oct 20;11:140. doi: 10.1186/1471-2229-11-140 (PMC3209458; doi:10.1186/1471-2229-11-140)

F2

*S. lycopersicum* (Anl27)

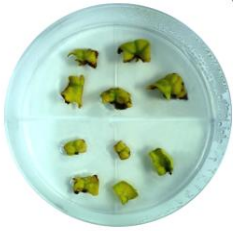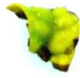

*S. pennellii* (PE-47)

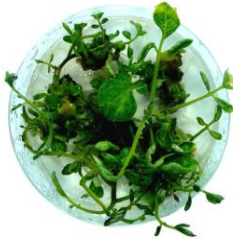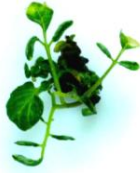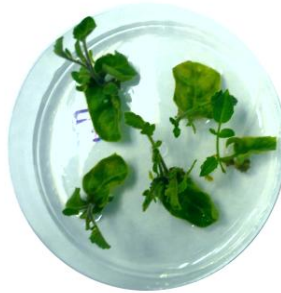

F1(Anl27xPE-47)

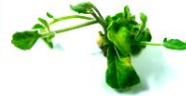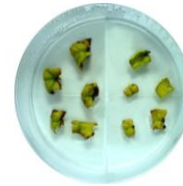

F2 45

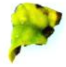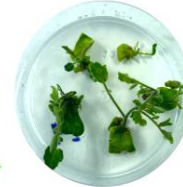

F2 60

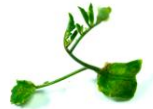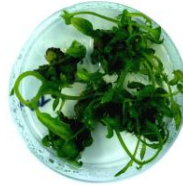

F2 53

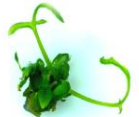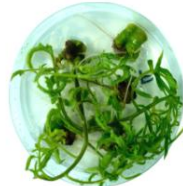

F2 66

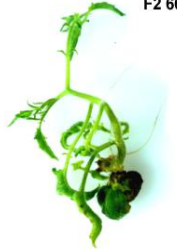

BC1

*S. lycopersicum* (Anl27)

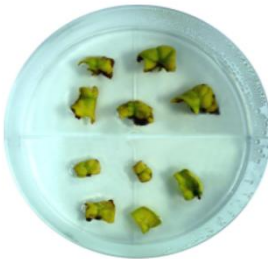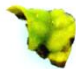

F1(Anl27xPE-47)

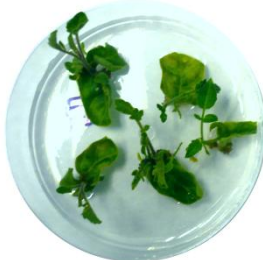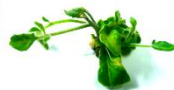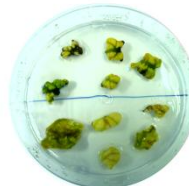

BC1 13/73

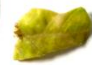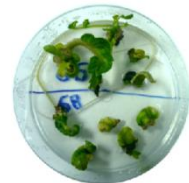

BC1 65/68

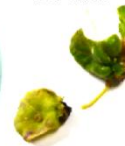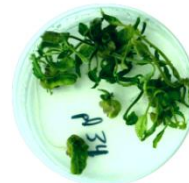

BC1 34

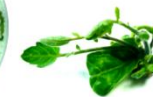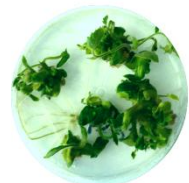

BC1 81

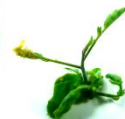

Supplement: Additional file 1 — Regeneration response of leaf explants. Regeneration response of leaf explants from parents [tomato (cv. Anl27); S. pennellii (PE-47)], F1, F2 and BC1 populations, cultured on shoot induction medium (SIM) for 30 days and transferred to basal medium (BM) for 20 days. [file 1471-2229-11-140-S1.PDF]

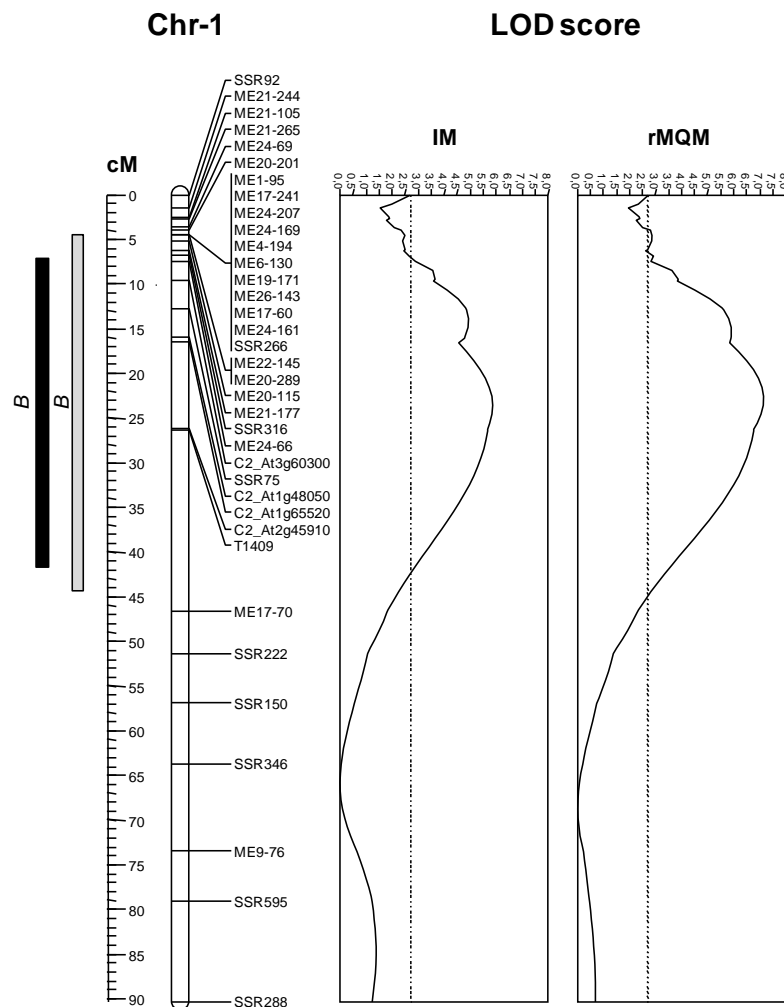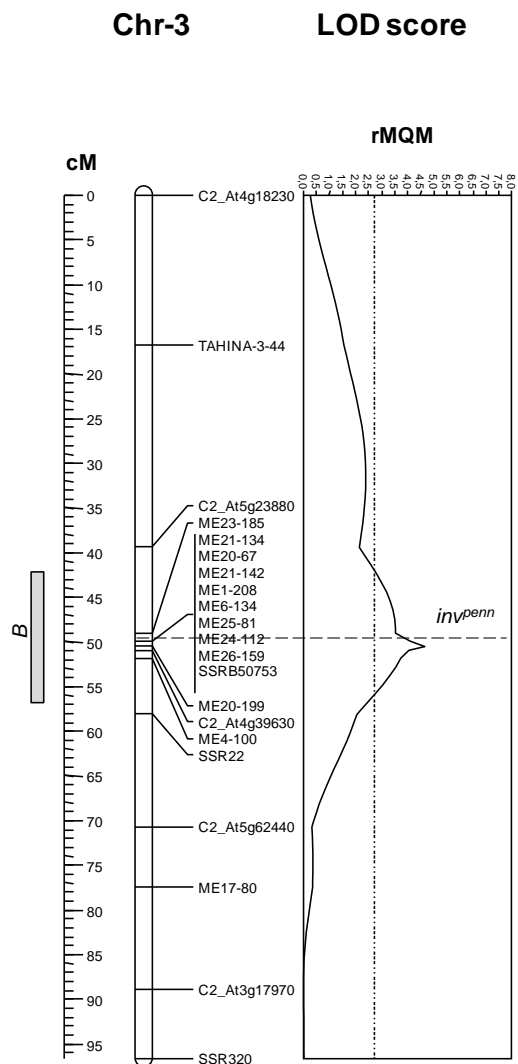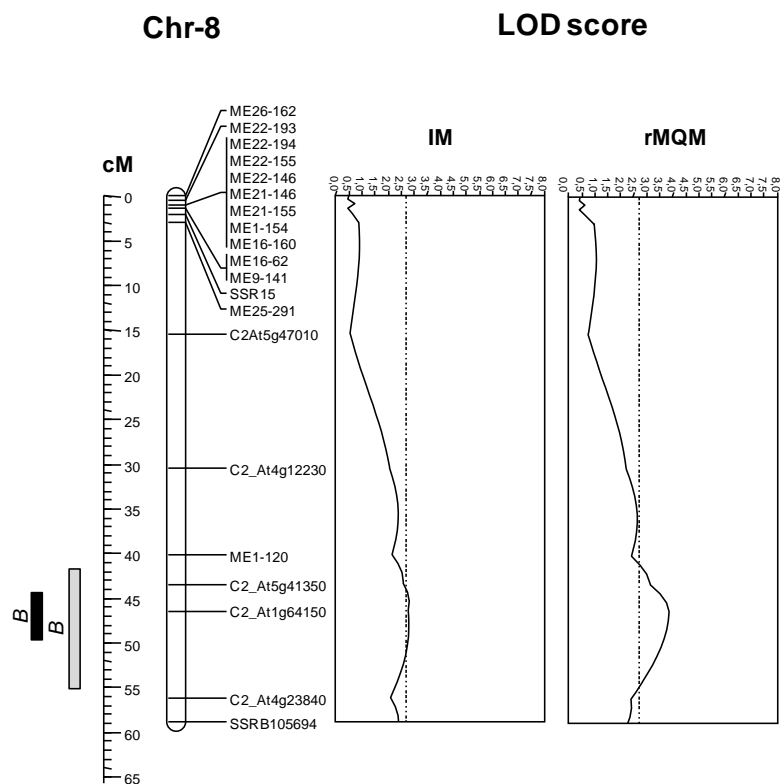

Supplement: Additional file 4 — Genetic location and LOD score profile of the BC1-QTLs for Bud percentage (B), detected on chromosomes 1 (SpRg-1), 3 (SpRg-3) and 8 (SlRg-8). Results from the Interval Mapping (IM) and restricted Multiple QTL Mapping (rMQM) approaches. On the left, projections as black bars (IM) and grey bars (rMQM) indicate the range of SpRg-1, SpRg-3 and SlRg-8 QTLs for B. The vertical dotted line indicates the 95% significant threshold value for declaring a QTL (B LOD threshold = 2.7). The horizontal dotted line indicates the position of the acid invertase gene (invpenn) marker included in the chromosome 3 QTL range. Map position (cM) and distances are based on the genetic linkage map developed in this study. [file 1471-2229-11-140-S4.PDF]

Chr-1

LOD score

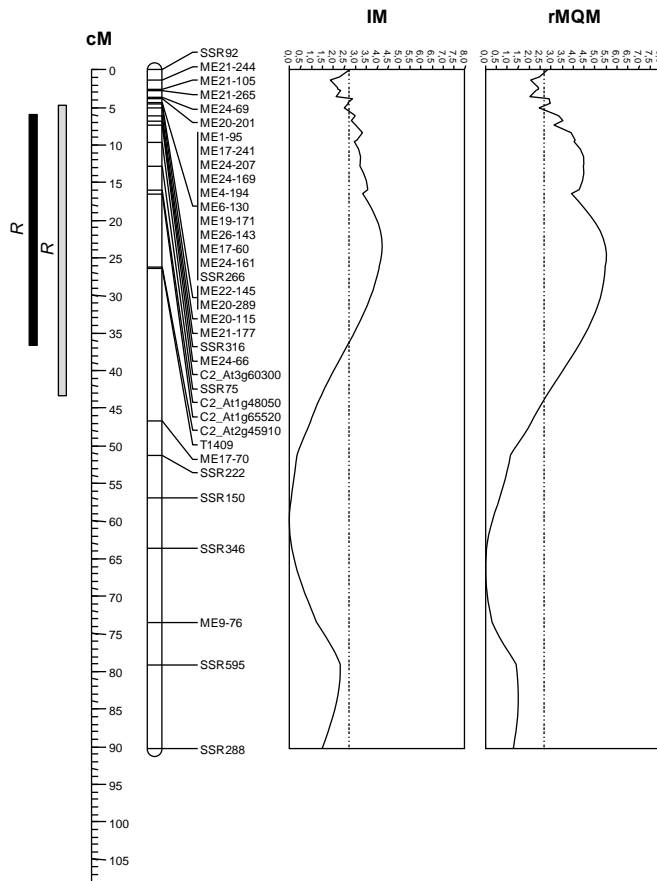

Chr-4

LOD score

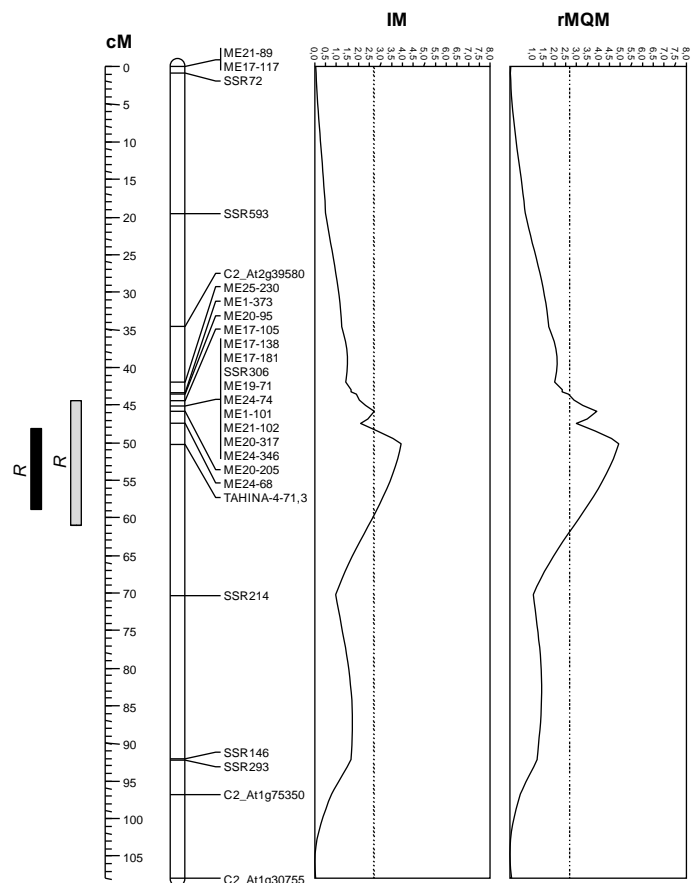

Chr-7

LOD score

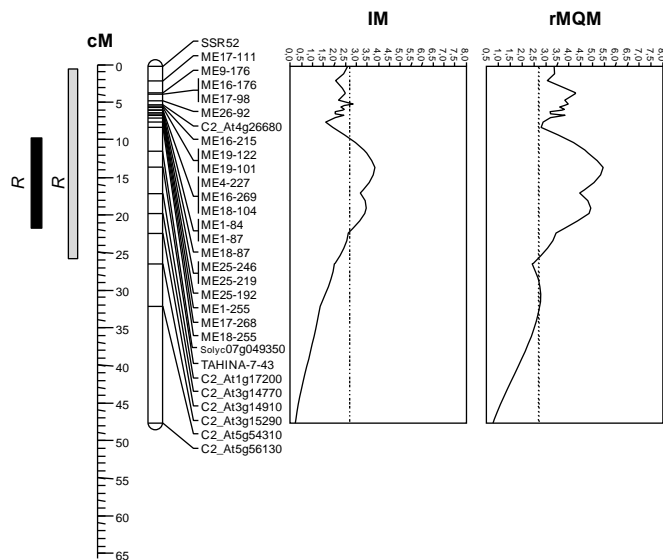

Chr-8

LOD score

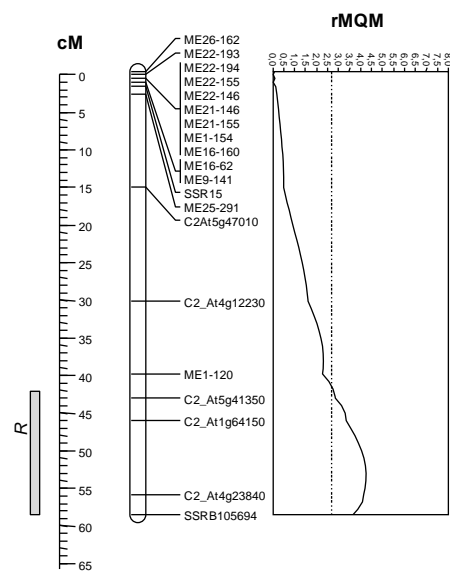

Supplement: Additional file 5 — Genetic location and LOD score profile of the BC1-QTLs for Regeneration percentage (R), detected in this study on chromosomes 1 (SpRg-1), 4 (SpRg-4a), 7 (SpRg-7) and 8 (SlRg-8). Results from the Interval Mapping (IM) and restricted Multiple QTL Mapping (rMQM) approaches. On the left, projections as black bars (IM) and grey bars (rMQM) indicate the range of SpRg-1, SpRg-4a, SpRg-7 and SlRg-8 QTLs for R. The vertical dotted line indicates the 95% significant threshold value for declaring a QTL (R LOD threshold = 2.7). Map position (cM) and distances are based on the genetic linkage map developed in this study. [file 1471-2229-11-140-S5.PDF]

Chr-1

LOD score

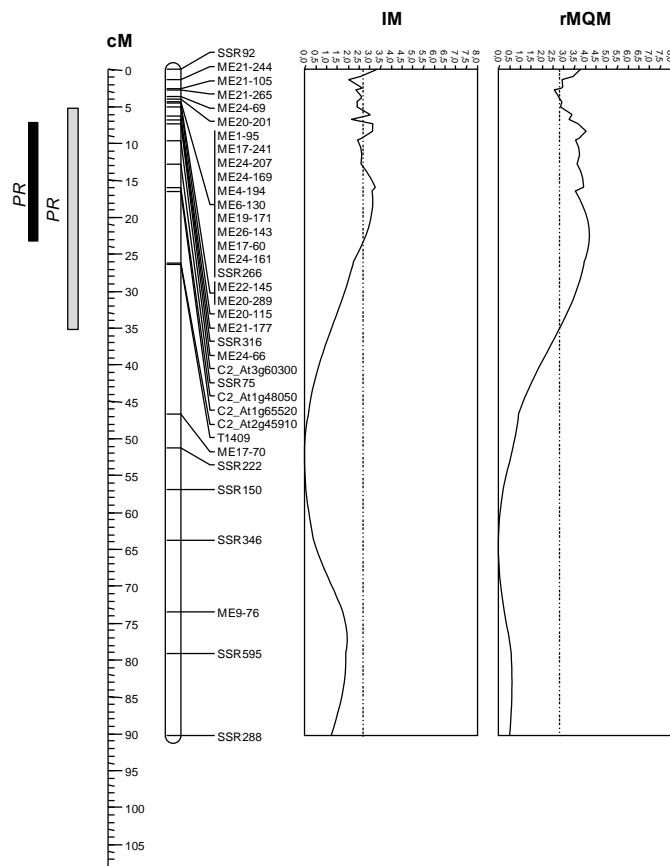

Chr-3

LOD score

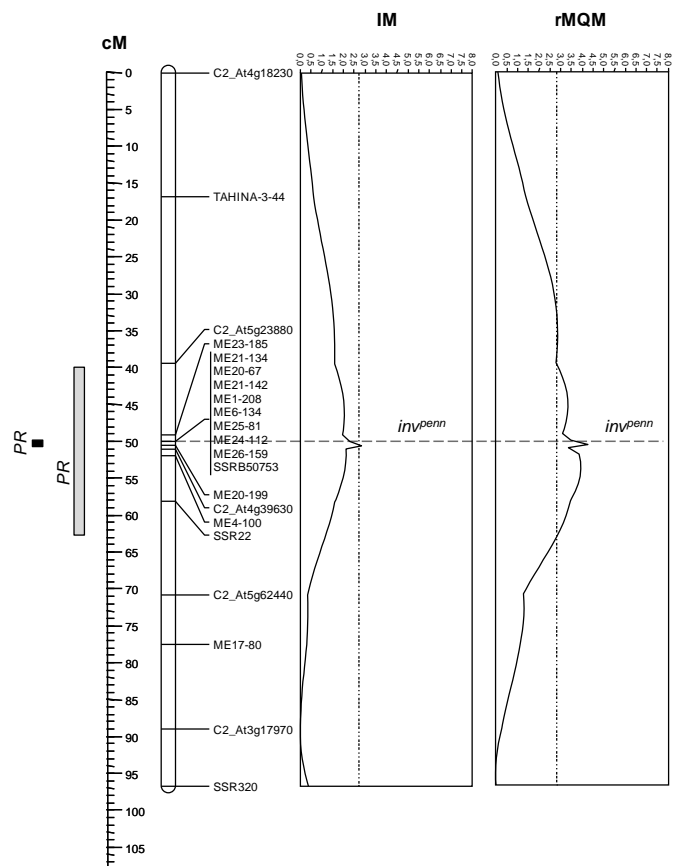

Chr-4

LOD score

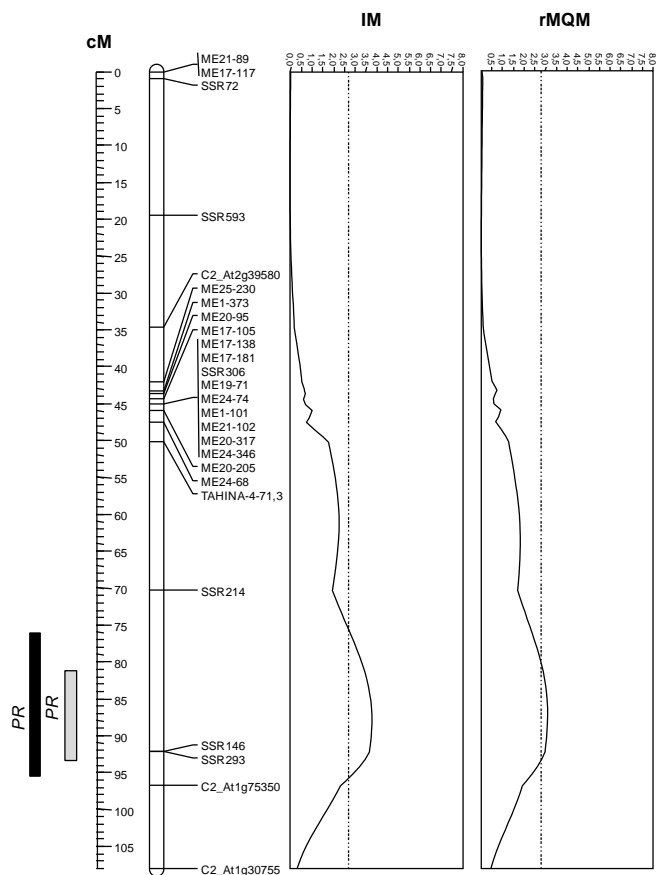

Chr-7

LOD score

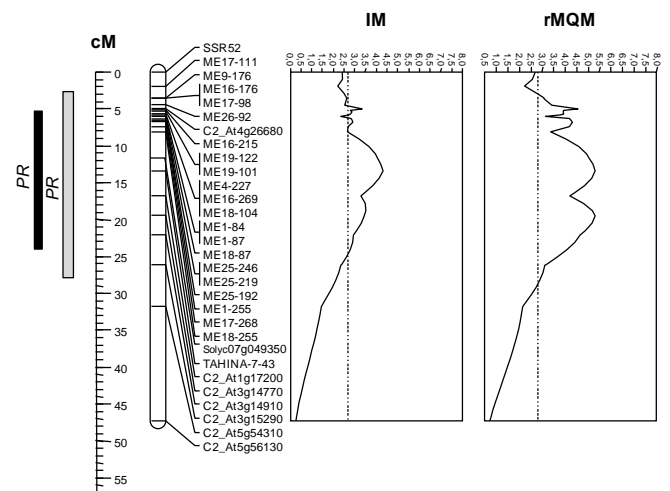

Supplement: Additional file 6 — Genetic location and LOD score profile of the BC1-QTLs for Productivity Rate (PR), detected in this study on chromosomes 1 (SpRg-1), 3 (SpRg-3), 4 (SpRg-4b) and 7 (SpRg-7). Results from the Interval Mapping (IM) and restricted Multiple QTL Mapping (rMQM) approaches. On the left, projections as black bars (IM) and grey bars (rMQM) indicate the range of SpRg-1, SpRg-3, SpRg-4b and SpRg-7 for PR. The vertical dotted line indicates the 95% significant threshold value for declaring a QTL (PR LOD threshold = 2.8). Horizontal dotted lines indicate the position of the acid invertase gene (invpenn) marker included in the chromosome 3 QTL range. Map position (cM) and distances are based on the genetic linkage map developed in this study. [file 1471-2229-11-140-S6.PDF]

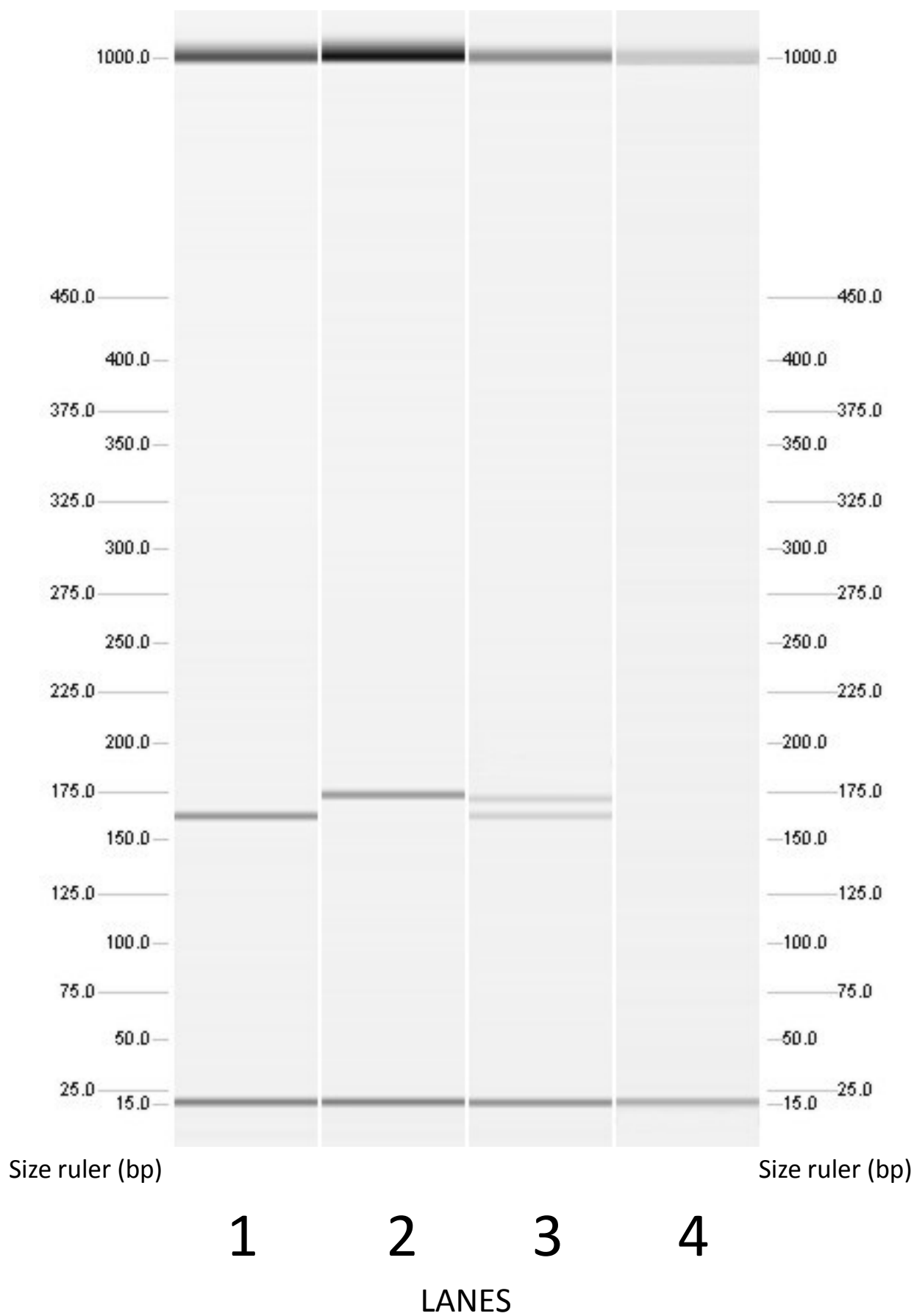

Supplement: Additional file 7 — Polymorphic acid invertase gene marker (invpenn). Amplified bands separated using the multicapillary electrophoresis QIAxcel System. Lane 1: S. lycopersicum L. (Anl27), band size (~162bp). Lane 2: S. pennellii PE-47, band size (~173bp). Lane 3: F1 Hybrid S. lycopersicum L. (Anl27) × S. pennellii PE-47, both bands (~162bp-~173bp). Lane 4: negative control. [file 1471-2229-11-140-S7.PDF]
