# Supplementary material for: Localization of QTLs for in vitro plant regeneration in tomato
Source: BMC Plant Biol. 2011 Oct 20;11:140. doi: 10.1186/1471-2229-11-140 (PMC3209458; doi:10.1186/1471-2229-11-140)
Supplement: Additional file 2 — In silico-designed SSR markers. Table with the name, band size, repeat motif, temperature of annealing and primers sequences of in silico-designed SSR markers. [file 1471-2229-11-140-S2.PDF]

Name[1]	Band size (bp)[2]	Repeat Motif[3]	Ta (C°)[4]		Sequence (5´–3´)
[b] TAHINA-2-92*	196	(TA)16	52	F	AGCGAATTATGCATGTGTGTG
				R	TTTTCTCGCGATAACTGTTTTG
[a] TAHINA-2-118	201	(AT)27	59	F	CAGAAGATTCGAAGGGTGCT
				R	AGGTGACTCCTCCACTGTCG
[d] TAHINA-2-139,5a	163	(AT)12	58	F	CCATACATGTTATAGGGAGGAGAG
				R	CCTCTCCCATTTTTGTTTGC
[a] TAHINA-2-139,5b	237	(TTA)10	50	F	ATGTGCACACACGTGAACC
				R	ATGCAATTGCCATTGACCTA
[a] TAHINA-3-30*	250	(TACA)10	53	F	ACCGGCGATTCTCTTGTTC
				R	GGCACAAATCCTAAGCAAAAC
[a] TAHINA-3-44	161	(TA)7(CA)10	55	F	TGGGTCGAGTCGGATATTTT
				R	CTAAAAGCGTGGGCTTCACT
[e] TAHINA-3-54,5*	220	(AT)21	55	F	AAACACCCTCTTAGTTTCAATCC
				R	CACCCCACCCAATATTTAAAAC
[a] TAHINA-3-59	150	(TA)11	54	F	ACGTATTGTGATGGTCATATACAGTT
				R	CACATAATCCCATGATTTAAGCAA
[a] TAHINA-3-110*	242	(ATA)5	55	F	AGAAGCGTACCCAATCATGC
				R	GGCTGGATTGTGAGTGGATT
[b] TAHINA-3-123	244	(AAT)11	55	F	GCGCTGAATCTCAAACTCG
				R	AGCAAAGGGGTTGTACATGG
[b] TAHINA-3-133	189	(TAT)8	53	F	AACCGCAGAAACGAAGAAAA
				R	GAAAATGGTTTACACAAAAGATGC
[b] TAHINA-3-137	152	(AT)24	58	F	CAAGTGATATATTTGGGGAGCTG
				R	GAACCTACATATCGTAGCCATCTTC
[c] TAHINA-3-141	221	(CT)5	55	F	CAAGAATGCACCAGTTCCAA
				R	TTGGCCCATACCATAGCTTC
[a] TAHINA-4-71,3	175	(TA)20	51	F	AAGTTTGTGCCGCAGCTTAG
				R	TGTGCAACCCATGTGTTTAT
[b] TAHINA-4-125*	166	(TA)13(ACAT)5	55	F	AGGGCTATGCCCATAGTGTG
				R	TTGATGACTTGGCAGTGTGG
[c] TAHINA-5-10	201	(TAA)3(AT)18	58	F	AAGCATAGTATCACAAAGCTGACC
				R	ATGGAAGCCACAACCAAATG
[c] TAHINA-5-51	192	(AT)24	59	F	TCAATGTTAGGACTTTAGGGGTTC
				R	AAAGCAGAGCCAGGTGAAAG
[b] TAHINA-5-60a	191	(AT)11	58	F	AGGGGCAAAACAGTAATTCAA
				R	GACTCTCTAAATATTGTGCAAGCCTA
[a] TAHINA-5-60b*	242	(CA)6	58	F	GAGTTTATCGCACCCTCACC
				R	GGGAGGTGTTCGAGTAGGAG
[c] TAHINA-5-77	154	(ACA)6	57	F	AAGTGCTCCCTTCCTCCAAT
				R	TCCCAAGACCCCAACCTAGT
[b] TAHINA-5-84a	202	(TA)7(GA)6(G)12	52	F	CATTCGAAAATAGAATAACTGGCTAA
				R	TGCGATCACTTTTTATTGCTG
[b] TAHINA-5-84b*	205	(TA)19	53	F	GAAGCTAAACTCTAAGAAACGTGTG
				R	CATGCAAATGGATGGAACAC
[e] TAHINA-6-18,5	284	(ATA)8	52	F	GCGATATTTTGCTTTTTCACTT
				R	AAAAATTATTATGAGATGCAAATCAAC
[e] TAHINA-6-59	250	(AT)15	53	F	TTTTCCTGGGGTAAGCAGAA
				R	TTCAACTTTTCACTTTGGAGCTT
[b] TAHINA-6-69	168	(AT)18	59	F	CTTGCAAATGAAGGGTCTCC
				R	AGGATTGGACCAGTGTTTTCA
[a] TAHINA-6-74*	248	(CA)11(TA)5(CA)4(TACA)2	55	F	TCCTTGTCACGATCGGGTAG
				R	TGTTGAGTCTAAGATGGCAAAAG
[a] TAHINA-6-85	229	(AT)9	55	F	CATGTTGGCCAAACAATCTG
				R	GCAAGGGATGCTGTCTTCTT
[e] TAHINA-6-92,5	287	(AT)27	59	F	TATGCCCAATCGAACACTTG
				R	AGGAACAAACGGGCATTTAG
[a] TAHINA-7-43	247	(AT)23	60	F	GCAGCCAAATAGAAATTGGAAG
				R	CACATGTTAAAAGGTTGGTCAC
[b] TAHINA-7-63,5a	250	(TA)29	59	F	AGATGTGGACCTCCTTCGAC
				R	TTCTCACCTAACCCAGTACCAC
[a] TAHINA-7-63,5b*	187	(TAT)7	53	F	GGCCAGCAATTATTAGAGTAGG
				R	TTACTAATTTGGCCGGCTTG
[e] TAHINA-7-72	158	(TA)11	52	F	TTATTTTTGTCTTCGCTTTATTTTT
				R	AACTCCCAAAGCGTAATTTGA
[a] TAHINA-7-73*	205	(ATT)7	50	F	GGAAGCTTGATTAGTGGAATGG
				R	TTTTTGGGAGAGCATGTTTG
[e] TAHINA-7-92a	221	(TTA)10	59	F	TCGAACTCCCAAAACGGATA
				R	CATTGAGGAAAAGGTTTGAGG
[e] TAHINA-7-92b*	182	(AT)24	55	F	GGGGCCTTCTAAACTCATCG
				R	TGGCCTACCTTGCTTTCTTC
[c] TAHINA-7-104a	244	(ATA)11	59	F	TGCCCTTAATTATGCGAACAG
				R	TGGCAATCTCTAGTGAAAATGTC
[a] TAHINA-7-104b*	239	(AT)29	52	F	TTGGACTAATCTAAAAGGCATTGAC
				R	TTTTGTAAATAATGGCAAATGTGAG
[a] TAHINA-8-2*	257	(TA)21	57	F	AAGCTAACAACATTAACTCAATTATCG
				R	TGGTTGGAGGTATTTATAGGGTTG
[b] TAHINA-8-71a	238	(TA)11	52	F	TCAAAACATGAATTTGCCTGA
				R	TGGATTTTGGATTGACTATTGC
[a] TAHINA-8-71b*	195	(AT)12	55	F	TCCAGGATAGACCTTGAGGAAC
				R	TCCACCAGCTTTTGGATCTG
[e] TAHINA-9-0a	414	(AT)37	59	F	TCCCCAATGAAGTCATTCAAC
				R	GAAAGGTCAAGATTAGGGAATGAG
[a] TAHINA-9-0b	232	(TA)21	52	F	AAAAGGTTCACGAAGGGAAAA
				R	AATCAAAACACCTTCAACGACT
[a] TAHINA-9-73*	222	(ATT)19	55	F	CAATGGGCAGGTACTCCATC
				R	GCACAGCAACATTACCAACAG
[a] TAHINA-9-90	254	(AT)37	58	F	GTCATGTTTGTCTTGATTCTCG
				R	TCCCTTCCTACCCTTTTTGG
[d] TAHINA-11-45	189	(TA)22	54	F	GAGAGGTACAAAAATAAGTGCAAAA
				R	AGACAAACAGAAAAGAAGACATGA
[b] TAHINA-11-53,5*	236	(AT)17	52	F	CGATAGCATTTGATCTGATTTTG
				R	AATTTTAACTGCATCGCATGG
[a] TAHINA-11-61	242	(TA)27(TG)17	53	F	AGTGACCAAGGCTGCCATAA
				R	AATCCTTCTTGGCTGGAATG
[c] TAHINA-11-74	246	(TA)7(GA)9	60	F	GCCATCGAAGAAATGGAAAA
				R	ACCTAAGGGCGGAGCTAGAG
[b] TAHINA-11-76	312	(AT)25	58	F	TAAACGAATCGGGACAGAAC
				R	CGTACACACATCAAACACTCACC
[b] TAHINA-11-91a	198	(AT)13	59	F	TGGACTCTCTCCAACTACTCACC
				R	GAGTTTGTTCATGCAATGTGG
[a] TAHINA-11-91b*	223	(A)12(GA)8(TAT)4	56	F	TGTGTACTGTTGCCTTTGCAG
				R	TCATAATCTCTATGGTCAAATGCTG
[c] TAHINA-11-91,5	224	(CT)9(TA)6	59	F	TTTGGTGGGGACTGACAAAT
				R	CGAAGCACGTATGCAAAAAC
[a] TAHINA-12-12,5a	227	(AT)22	58	F	CATCACACTCAGACCCCACT
				R	GGGGGTTTAAAATCCACCTT
[a] TAHINA-12-12,5b	262	(TAA)14(CAA)6	52	F	GGCTGCATAAAAATTCGGTTA
				R	AAACAGTAATGACTTTTGAATTTTCTT
[a] TAHINA-12-39	192	(TTA)26	59	F	ATTGCCACGTGGATTGACTC
				R	TGCAAGCTGTTCTTTTCAGAC
[b] TAHINA-12-71	236	(AT)35	58	F	CCTCGACATGACAAATCACA
				R	CAGAAATAGTGGAATGGGATCA
[b] TAHINA-12-85,7	338	(TA)10(AG)9	58	F	TTACCAGGGGTAGGAGTTGG
				R	TGGTCTTCTACATACATCAACTGAA
[c] TAHINA-12-97*	197	(AAT)7	54	F	ACAAGGCAAATTGGATGAGG
				R	AAAATGAGAGAAGGAAAAGGAAAG
[e] TAHINA-12-116	209	(TAA)19(GAA)8	58	F	TTTTTCAACTGTGAGGAAAATTG
				R	CATTATTTTCTTCATTTTACTTTGCTC
[b] TAHINA-12-120	150	(AT)36	59	F	AAGATAGCTGGGCCTTTGGT
				R	ACTCTCTCTCACACACGCACA

[1] Name is composed by the word TAHINA followed by the number of chromosome and the BAC position
[2] PCR predicted band sizes for *S. lycopersicum*
[3] Number indicates motif repetitions
[4] Annealing temperature

[a] Codominant polymorphic markers
[b] Dominant polymorphic markers
[c] Monomorphic markers
[d] Multiple bands
[e] No amplification

* Designed by "Aprovechamiento de la variabilidad extraespecífica en la mejora del tomate" COMAV research group
