# Supplementary material for: Localization of QTLs for in vitro plant regeneration in tomato
Source: BMC Plant Biol. 2011 Oct 20;11:140. doi: 10.1186/1471-2229-11-140 (PMC3209458; doi:10.1186/1471-2229-11-140)
Supplement: Additional file 3 — Genetic location and LOD score profile of the F2-QTLs for regeneration components detected by Interval Mapping on chromosome 7 (SpRg-7). Genetic location and LOD score profile of the F2-QTLs for regeneration components (Bud percentage (B), Regeneration percentage (R) and Productivity Rate (PR)). On the left, projections of QTLs as black bars indicate the SpRg-7 for B, R and PR traits. The vertical dotted line indicates the 95% significant threshold value for declaring a QTL (B LOD threshold = 3.7) (R LOD threshold = 3.6) (PR LOD threshold = 4.4). Map position (cM) and distances are based on the genetic linkage map developed in this study. QTLs characteristics in attached table. [file 1471-2229-11-140-S3.PDF]

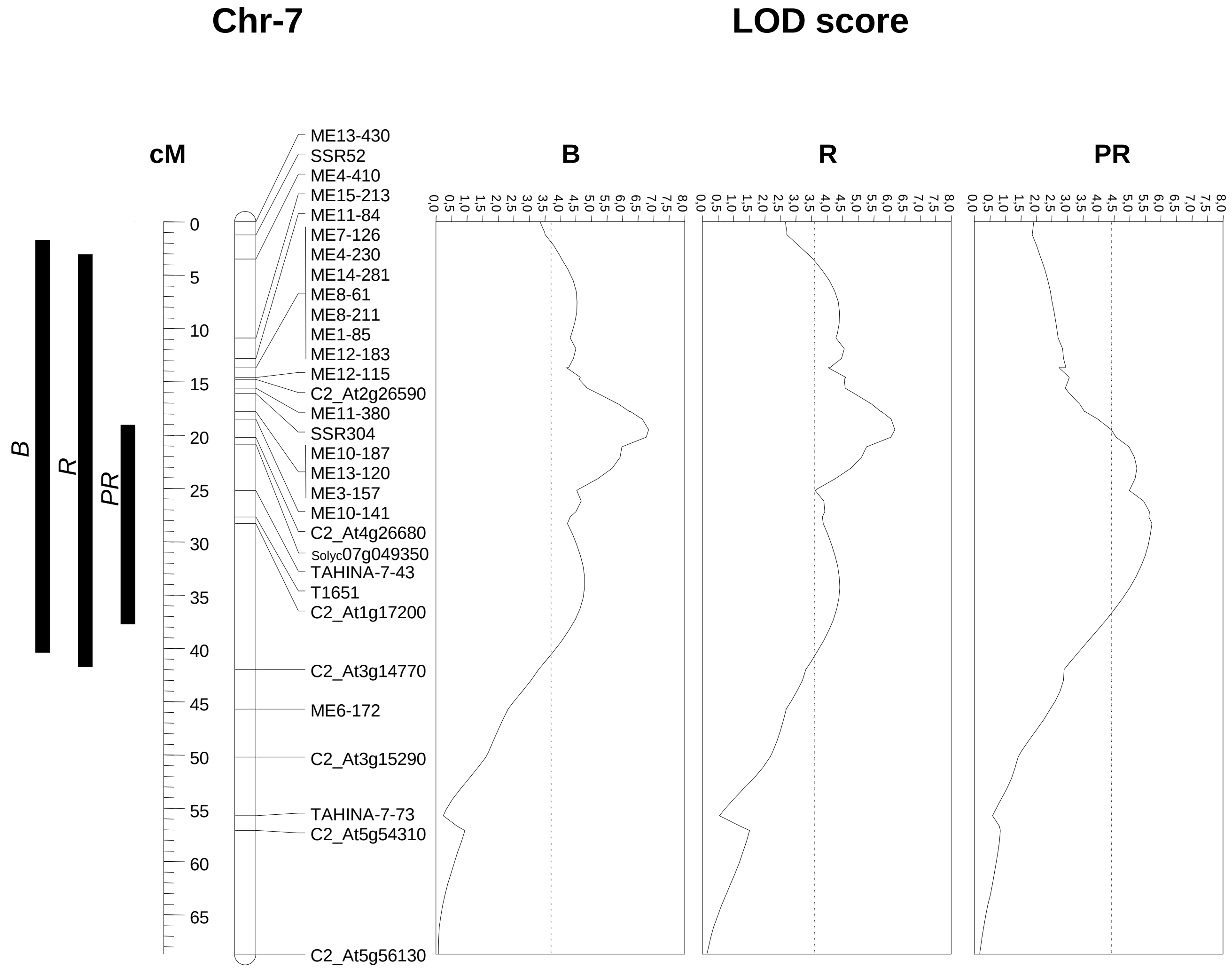

QTL analysis	Trait	QTL	Genome wide significant threshold level (P < 0.05)	Chr	Start(cM)	Finish(cM)	Coverage (cM)	LOD Peak	Position of LOD peak (cM)	Peak marker [a]	%variance explained	Estimated additive effect	Estimated dominance effect
IM	B	*SpRg-7*	3.7	7	2.20	40.28	38.08	6.84	19.51	ME10-141/C2_At4g26680	27.0	-22,20	12,32
IM	R	*SpRg-7*	3.6	7	4.50	40.28	35.78	6.18	19.51	ME10-141/C2_At4g26680	24.8	-23,29	13,63
IM	PR	*SpRg-7*	4.4	7	19.51	36.28	16.77	5.72	28.28	C2_At1g17200	23.1	-1,53	-0,55

[a] In case of the absence of a peak marker, loci flanking the likely peak of a QTL are shown.
