# Supplementary material for: Localization of QTLs for in vitro plant regeneration in tomato
Source: BMC Plant Biol. 2011 Oct 20;11:140. doi: 10.1186/1471-2229-11-140 (PMC3209458; doi:10.1186/1471-2229-11-140)
Supplement: Additional file 8 — Markers used for genotyping the F2 and BC1 population. SSR, COS, COSII, CAP markers used for genotyping the F2 and BC1 population. [file 1471-2229-11-140-S8.PDF]

	SSR		COSII		COS		CAP	
Chr	F2	BC1	F2	BC1	F2	BC1	F2	BC1
1	SSR92; SSR266; SSR316; SSR75; SSR222; SSR150; SSR288	SSR92; SSR266; SSR316; SSR75; SSR222; SSR150; SSR346; SSR595; SSR288	C2_At2g45910	C2_At3g60300; C2_At1g48050; C2_At1g65520; C2_At2g45910		T1409		
2	SSR586; SSR356; SSR5; SSR26; TAHINA-2-118; TAHINA-2-139,5b	SSR586; SSR66; SSR5; SSR26; TAHINA-2-118; TAHINA-2-139,5b						
3	TAHINA-3-30; SSR22; SSR320; SSR601	TAHINA-3-44; SSRB50753; SSR22; SSR320	C2_At4g18230; C2_At5g62440; C2_At3g17970	C2_At4g18230; C2_At5g23880; C2_At4g39630; C2_At5g62440; C2_At3g17970				
4	SSR72; SSR593; SSR306; TAHINA-4-71,3; SSR214; SSR293	SSR72; SSR593; SSR306; TAHINA-4-71,3; SSR214; SSR146; SSR293	C2_At1g30755	C2_At2g39580; C2_At1g75350; C2_At1g30755				
5	SSR325; SSR602; SSR115; TAHINA-5-60b; SSR49; SSRB18031	SSR325; SSR602; SSR115; SSR49; SSRB18031	C2_At1g60440; C2_At3g26085; C2_At1g10500	C2_At4g24830; C2_At3g26085; C2_At1g10500				
6	SSR48; SSR578	SSR48; SSR578	C2_At5g62530; C2_At1g12060; C2_At1g18640; C2_At4g03180	C2_At5g62530; C2_At1g12060; C2_At1g18640; C2_At4g03180	T0507	T0507		
7	SSR52; SSR304; TAHINA-7-43; TAHINA-7-73	SSR52; TAHINA-7-43	C2_At2g26590; C2_At4g26680; C2_At1g17200; C2_At3g14770; C2_At3g15290; C2_At5g54310; C2_At5g56130	C2_At4g26680; C2_At1g17200; C2_At3g14770; C2_At3g14910; C2_At3g15290; C2_At5g54310; C2_At5g56130	T1651		Solyc07g049350	Solyc07g049350
8	SSR15; TAHINA-6-74; SSR63; SSRB105694	SSR15; SSRB105694	C2_At1g18480; C2_At5g47010	C2_At5g47010; C2_At4g12230; C2_At5g41350; C2_At1g64150; C2_At4g23840				
9	SSR73; SSR383; TAHINA-9-90; SSR599	SSR73; SSR383; TAHINA-9-90; SSR599	C2_At5g02740; C2_At1g07310	C2_At5g02740; C2_At1g07310				
10	SSRB102358; SSR248; SSR85; SSR223	SSR85; SSR223	C2_At5g60990; C2_At4g04930	C2_At4g04930			TG230; TG303; TG233	TG233
11	SSR136; SSR80; SSR46; TAHINA-11-61	SSR136	C2_At2g22570; C2_At5g20890; C2_At2g28490; C2_At5g59960	C2_At2g22570; C2_At5g20890; C2_At2g28490; C2_At5g59960				
12	TAHINA-12-12,5a; TAHINA-12-39	TAHINA-12-12,5a; TAHINA-12-39	C2_At3g25910; C2_At1g06550; C2_At3g24490; C2_At2g25740; C2_At1g48300; C2_At5g21170	C2_At3g25910; C2_At1g06550; C2_At2g25740; C2_At1g48300; C2_At5g21170	T0801	T0801		
